# Expression of Spindle and Kinetochore-Associated Protein 1 Is Associated with Poor Prognosis in Papillary Thyroid Carcinoma

**DOI:** 10.1155/2015/616541

**Published:** 2015-05-04

**Authors:** Chao Dong, Xiao-li Wang, Bin-lin Ma

**Affiliations:** Department of Head and Neck Surgery, Tumor Hospital Affiliated to Xinjiang Medical University, Urumqi, Xinjiang 830011, China

## Abstract

*Aim*. Spindle and kinetochore-associated protein 1 (SKA1) is one subtype of SKA, whose protein can make spindle microtubules attach steadily to the kinetochore in the middle of mitosis. At present, there are fewer researches on the relationship between SKA1 expression and tumor development. *Methods*. In this study, immunohistochemical analysis was used to determine the expression of SKA1 in papillary thyroid carcinoma (PTC) and adjacent tissues. We used quantitative real-time polymerase chain reaction (qRT-PCR) and Western blot analysis to further verify the results. *Results*. We found that SKA1 expression was significantly higher in PTC tissues than normal adjacent tissues (*P* < 0.05). There existed a significant correlation among a higher SKA1 expression, including lymphoid node (*P* = 0.005), clinical stage (*P* = 0.015), and extrathyroid invasion (*P* = 0.004). Survival analysis showed high SKA1 expression in PTC patients more likely to relapse after surgery. *Conclusion*. High SKA1 expression is predictive of poor prognosis of PTC, implying that SKA1 may be a promising new target for targeted therapies for PTC.

## 1. Introduction

Thyroid cancer is the most common malignant tumor of the endocrine system and the most common head and neck tumor. Each year, new cases of thyroid cancer account for 1–5% of all cancer cases [1]. Over the past 30 years, a rapid increase in the incidence of thyroid cancer has attracted widespread attention [2]. Thyroid cancer can be classified as papillary thyroid carcinoma (PTC), follicular thyroid carcinoma (FTC), medullary thyroid carcinoma (MTC), undifferentiated thyroid carcinoma (ATC), and others according to its pathological characteristics; PTC is the most common type, accounting for 79–94% of thyroid cancer cases [1]. With comprehensive surgery-focused antineoplastic therapy, the prognosis of most patients with PTC is good, but 20% these patients relapse after treatment and require reoperation [3]. Consequently, analyzing the biological characteristics of PTC and exploring new targets for therapy have been major clinical concerns.

It is well known that, during the cell cycle, the proper formation and depolymerization of the spindle play an essential role in maintaining normal mitosis of eukaryotic cells. However, chromosomal division abnormalities can cause genetic instability and ultimately induce PTC development [4]. Spindle and kinetochore-associated protein 1 (SKA1) is a subtype of SKA that causes spindle microtubules to attach firmly to the kinetochore in the middle of mitosis [5]. At present, few research studies have examined the relationship between SKA1 expression and tumor development. In our study, we use immunohistochemistry to observe SKA1 expression in PTC, and we conducted a quantitative analysis of SKA1 using quantitative real-time PCR and Western blotting with the aim of investigating the association between SKA1 and the prognosis of patients with PTC.

## 2. Materials and Methods

### 2.1. Patients and Samples

The subjects of this study were 123 patients with PTC who had undergone surgical intervention at the Department of Head and Neck Surgery of the Affiliated Tumor Hospital of Xinjiang Medical University between 2005 and 2009. All patients did not receive any therapy prior to surgery and were diagnosed with primary PTC based on the pathological findings. All 123 patients had complete clinical data, including age, sex, tumor size, lymph node status, clinical stage, and extrathyroidal invasion. Patients were selected for this study only if follow-up examinations and clinical data were available. The TNM stage was defined according to the 7th edition of the TNM classification of the International Union Against Cancer. The study was approved by the ethics committee of the Affiliated Tumor Hospital of Xinjiang Medical University. Written informed consent was obtained from each patient for the use of resected samples for research.

### 2.2. Immunohistochemistry

Biopsy tissue samples were sectioned into 4-*μ*m slices and embedded with paraffin after fixation with 10% formaldehyde. Immunohistochemistry with the SP method (rabbit anti-SKA1 antibody; 1 : 500; Sigma Chemical Co., St. Louis, MO, USA) was used for testing by applying PBS instead of primary antibodies as the negative control, according to the manufacturer's instructions. The results were determined by two independent pathologists using a double-blind method. Five high power fields (200x) were randomly selected, and in each field 200 tumor cells were recorded, totaling to 1000 cells. According to the staining intensity, the cells were assigned the following point values: 0 points for no staining; 1 point for pale yellow staining; 2 points for brown-yellow staining; and 3 points for dark brown staining. The proportion of positive cells was assigned a value between 0% and 100%. The immunohistochemistry expression level was determined according to the product of the staining intensity and the proportion of positive cells. When the product was below 150%, this was considered low expression of SKA1, and when the product was above 150%, this was considered high expression of SKA1.

### 2.3. RNA Extraction and Quantitative Real-Time PCR Assays

The tissue samples were removed from liquid nitrogen, and RNA was extracted using Trizol reagent and a reverse transcription kit (Invitrogen; Carlsbad, CA, USA). The PCR primers used were SKA1-F: 5′-TGATGTGCCAGGAAGGTGAC-3′, SKA1-R: 5′-CAAAGGATACAGATGAACAACAGC-3′, GAPDH-F: 5′-GTGGACATCCGCAAAGAC-3′, and GAPDH-R: 5′-AAAGGGTGTAACGCAACTA-3′. All primers were synthesized by Shanghai Yingjun Biotechnology Company (Shanghai, China). The PCR reaction conditions were as follows: initial denaturation for 2 min at 94°C, 35 cycles of denaturation for 30 s, annealing for 30 s at 94°C, and extension for 1 min at 72°C, followed by 10 min at 72°C. PCR reaction product (3 *µ*L) was used for 2% agarose gel electrophoresis, and the grayscale analysis was performed with Quantity One software (Bio-Rad; Hercules, CA, USA). The relative expression level of* SKA1* mRNA was determined by the ratio of the grayscale value of* SKA1* mRNA and that of internal reference* GAPDH* mRNA.

### 2.4. Western Blot Analysis

After protein extraction using total protein lysis buffer, the protein concentration was determined by the BCA method. In brief, 80 *µ*g protein per well was used for 8% SDS-PAGE, and after electrophoretic separation, the protein was wet-transferred to polyvinylidene fluoride; 5% nonfat milk powder solution was added, and after a 1 h incubation at room temperature, the transferred protein was incubated with the primary antibodies (1 : 500 dilution SKA1 and GAPDH; Sigma Chemical Co., St. Louis, MO, USA) at 4°C overnight. Goat anti-rabbit IgG secondary antibody (GenScript Biotechnology Company, Nanjing, Jiangsu, China) marked with horseradish peroxidase and diluted at 1 : 1000 was added and incubated at room temperature for 1.5 h. The membrane was washed three times in TBST. The relative level of SKA1 protein was determined by the grayscale value of various protein straps and the ratio of the grayscale value of SKA1 to GAPDH protein.

### 2.5. Statistical Analysis

The chi-squared test was used to analyze the associations between SKA1 expression and the clinicopathological features of PTC.* SKA1* mRNA expression in PTC was compared with expression in normal adjacent tissues using the Mann-Whitney test (for 2 groups) or the Kruskal-Wallis test (for more than 2 groups). Kaplan-Meier analysis was used for survival analysis, lymph node recurrence-free survival (LNRFS), and distant recurrence-free survival (DRFS) calculations. LNRFS was defined as the time from the date of surgery to the date of lymph node relapse, and DRFS was defined as the time from the date of surgery to the date of distant recurrence [6, 7]. Cox regression models were used for multiple factor analysis. All statistical tests were two-tailed, and statistical significance was assumed for *P* < 0.05.

## 3. Results

### 3.1. Expression of SKA1 in PTC Tissues

**Figure 1:**
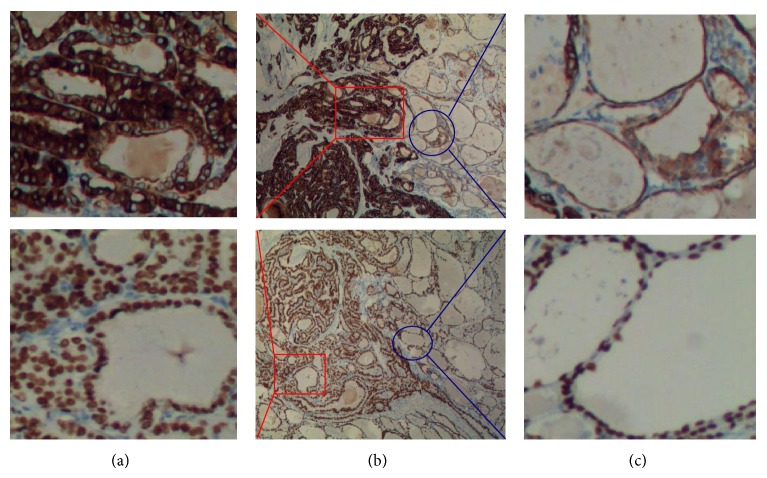
SKA1 expression by immunohistochemistry (SP × 10). Left side is PTC tissue (SP × 40); right side is normal adjacent tissue (SP × 40).

**Figure 2:**
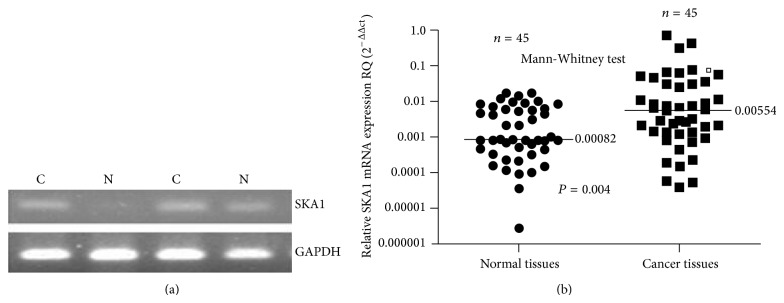
SKA1 mRNA was detected by qRT-PCR (N: normal tissues; C: cancer tissues).

**Figure 3:**
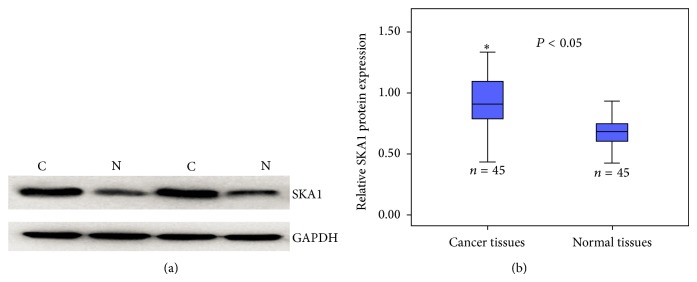
SKA1 protein was detected by Western blot analysis (N: normal tissues; C: cancer tissues).

Immunostaining results suggested a significantly higher SKA1 expression in PTC tissues compared with adjacent tissues. Among 123 tumor samples, 56 (45.5%) showed high SKA1 expression, with only 37% of the samples from normal adjacent tissues (see Figure 1). To confirm these observations, we investigated* SKA1* mRNA expression in 45 cases of PTC and normal adjacent tissues using qRT-PCR and Western blot analysis. The mRNA levels between PTC tissues and normal adjacent tissues were calculated with the comparative Ct method (2^−ΔΔct^). The results indicated that* SKA1* mRNA and SKA1 protein expression was significantly higher in PTC tissues than in normal adjacent tissues (*P* < 0.05, Figures 2 and 3).

### 3.2. Patient Characteristics

Table 1 shows the patients' clinical and immunohistochemical data. The mean age was 41.3 ± 15.7 years (range 26–69 years) for 32 male patients and 44.2 ± 14.5 years (range 24–73 years) for 35 female patients. Sixty-two patients (50.4%) had lymph node metastasis. Eighty-four patients (68.3%) had stage I-II disease based on the TNM staging system, and the remaining 39 patients (31.7%) had stage III-IV disease. During the median follow-up time, which was 52 months (range 17–102 months), 21 of 123 patients (17.1%) suffered lymph node recurrences, and 14 patients (11.3%) had distant organ recurrences. As shown in Table 1, there was a significant association between higher SKA1 expression and lymph node involvement (*P* = 0.005), clinical stage (*P* = 0.015), and extrathyroidal invasion (*P* = 0.004); however, no association was found between SKA1 expression and other clinicopathological features. According to the data, SKA1 expression in tumors might be useful for identifying the degree of PTC.

### 3.3. Expression of SKA1 and Prognosis of PTC Patients

**Figure 4:**
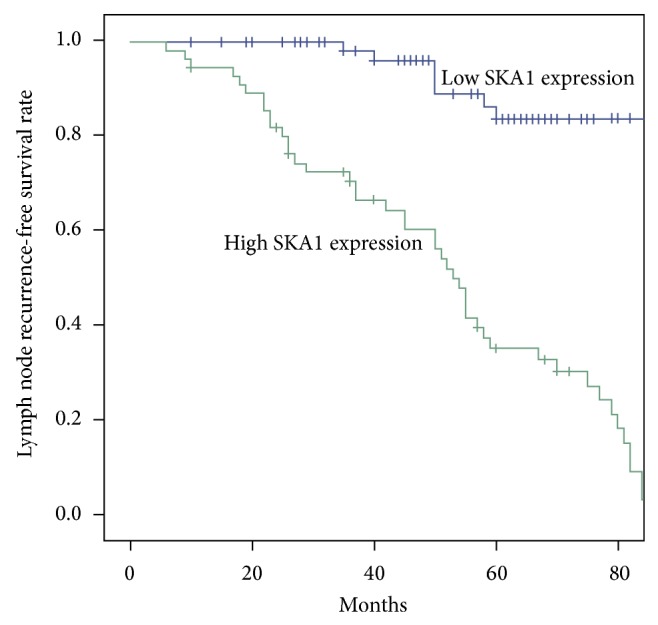
SKA1 expression and LNRFS.

**Figure 5:**
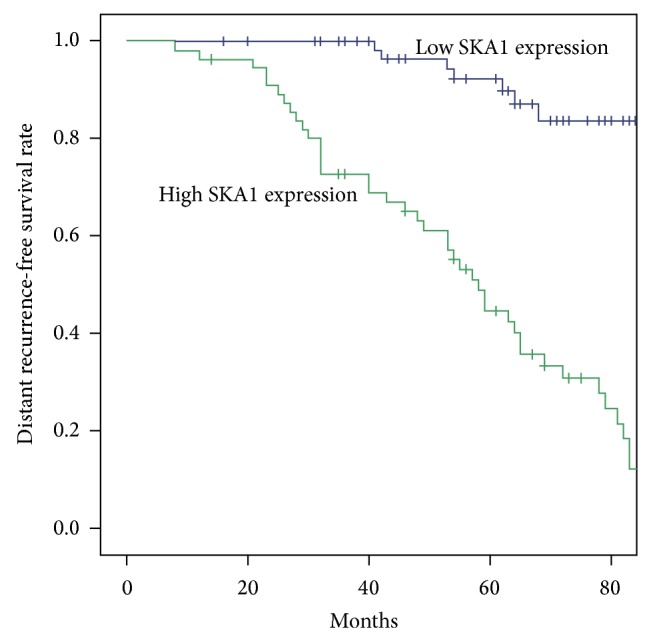
SKA1 expression and DRFS.

We used the Kaplan-Meier method to analyze the relationship between SKA1 expression levels and the prognosis of patients with PTC. The results showed that high SKA1 expression was associated with LNRFS (*P* < 0.001) and DRFS (*P* < 0.001) (Table 2, Figures 4 and 5). We performed multivariate analysis using the Cox regression model. The results of multivariate analysis for the mentioned parameters showed that high expression of SKA1 was an independent indicator of LNRFS and DRFS (Table 3).

## 4. Discussion

An equal distribution of chromosomes depends on the precise regulation of mitosis during each cell cycle, and the correct formation and depolymerization of the spindle are important for the regulation process [8]. Abnormalities of mitosis can lead to aneuploidy and genomic instability, ultimately resulting in cell death or tumorigenesis [9, 10]. The spindle, a temporary organelle directly related to cell division and chromosome movement, is composed of microtubules and conjugated proteins, and the kinetochore, a large protein complex, is an important link between mitotic chromosomes and spindle microtubules [11]. Based on one significant assumption, spindle orientation defects can lead to cell number increase by suppressing the asymmetric division of stem cells while promoting their symmetric proliferation [12, 13]. Furthermore, tissue architecture disorganization, a typical feature of malignant transformation, might occur due to defective spindle orientation [12, 14].

The* SKA1* gene is located on chromosome 18q21.1, contains 255 amino acids, and is approximately 30 kDa in size [15]. SKA1 is a necessary component for a stable kinetochore and microtubule binding, and it might = promote the combination of kinetochore and tubulin and regulate the depolymerization of tubulin and the synchronization of chromosomal migration and movement towards the 2 poles [16]. The SKA1 protein might be expressed not only in the spindle but also in the kinetochore [17]. Its carboxyl terminus contains a conserved microtubule-binding domain with multiple phosphorylation sites that can be phosphorylated by Aurora B kinase to further regulate the combination of SKA1 and microtubules [18]. Formation of the SKA complex helps to ensure the correct positioning of the spindle and kinetochore to promote the transition from middle to late mitosis.

Li et al. [19] found that SKA1 was overexpressed in human prostatic intraepithelial neoplasia by immunohistochemistry and quantitative RT-PCR. They also carried out in vitro experiments, which showed that prostate-specific upregulation of* SKA1* in a transgenic mouse model resulted in spontaneous tumorigenesis [19]. In addition, SKA1 is a critical factor that plays an essential role in the regulation of hepatocellular carcinoma cell proliferation and apoptosis; research showed that knockdown of* SKA1* inhibited hepatocellular carcinoma cell proliferation by inducing cell cycle arrest in the G0/G1 phase [20]. The few studies on this indicate that abnormal SKA1 expression might be closely associated with the development of other solid tumors, such as gastric cancer, oral adenosquamous carcinoma, and neuronal glioblastoma [5, 21, 22]. In this study, we found that SKA1 expression was associated with the clinicopathological features and prognosis of patients with PTC. Our results suggest that a high expression of SKA1 might be associated with lymph node status, clinical stage, and extrathyroidal invasion, further implying an involvement of SKA1 in the incidence and development of PTC. In addition, we demonstrated an association between high SKA1 expression and the prognosis of patients with PTC using Kaplan-Meier survival curves. Our findings indicate that a high expression of SKA1 indicates worse LNRFS and DRFS. At the same time, our multivariate analysis using Cox proportional hazards regression model showed that high expression of SKA1 was related to PTC patient prognosis. Based on these results, SKA1 might be an important prognostic indicator for PTC.

To the best of our knowledge, this is the first report on the relationship between SKA1 and prognosis in patients with PTC. High SKA1 expression is predictive of poor prognosis in PTC, implying that SKA1 might be a promising new target for targeted therapies for PTC. However, to determine the mechanism by which SKA1 upregulated expression promotes PTC generation and development and whether SKA1 also has abnormal expression in other solid tumors, further study is needed.

## Figures and Tables

**Table 1:** Correlation of high SKA1 expression with clinicopathologic characteristics of PTC patients.

Clinical features	SKA1 expression		*χ* ^2^	*P* value
	Low (*n* = 67)	High (*n* = 56)		
Age				
<45 years	22	15	0.531	0.466
≥45 years	45	41		
Sex				
Male	32	23	0.552	0.457
Female	35	33		
Hashimoto's thyroiditis				
Without	64	51	0.994	0.467
With	3	5		
Tumor size				
<2 cm	42	26	1.008	0.3799
≥2 cm	25	30		
Multifocality				
Without	43	34	0.156	0.712
With	24	22		
Differentiation				
Well	19	17	0.059	0.808
Poor	48	39		
Lymphoid node				
Without	41	20	7.922	0.005
With	26	36		
Clinical stage				
I-II	52	32	5.902	0.015
III-IV	15	24		
Extrathyroid invasion				
Without	46	24	8.280	0.004
With	21	32		

**Table 2:** LNRFS and DRFS of PTC patients with high and low SKA1 expressions.

	*N*	Survival time (months)	*P* value
		M ± SE	
*LNRFS *			
SAK1 low expression	67	84.31 ± 2.34	<0.001
SAK1 high expression	56	51.96 ± 3.38	
*DRFS *			
SAK1 low expression	67	87.14 ± 2.06	<0.001
SAK1 high expression	56	56.25 ± 3.23	

**Table 3:** Multivariate analysis of the prognosis of PTC.

Variable	LNRFS	*P* value	DRFS	*P* value
	HR for death (95% CI)		HR for death (95% CI)	
Lymphoid node	1.899 (1.054–3.422)	0.033	2.089 (1.144–3.817)	0.017
Clinical stage	2.827 (1.485–5.384)	0.002	3.026 (1.573–5.822)	0.001
Extrathyroid invasion	2.275 (1.197–4.324)	0.012	2.490 (1.305–4.751)	0.006
SKA1 high expression	4.862 (2.071–11.412)	<0.001	4.907 (2.099–11.475)	<0.001
